# Enhancing medical coding efficiency through domain-specific fine-tuned large language models

**DOI:** 10.1038/s44401-025-00018-3

**Published:** 2025-05-01

**Authors:** Zhen Hou, Hao Liu, Jiang Bian, Xing He, Yan Zhuang

**Affiliations:** 1 Department of Biomedical Engineering and Informatics, Luddy School of Informatics, Computing, and Engineering, Indiana University, Indianapolis, IN USA; 2 School of Computing, College of Science and Mathematics, Montclair State University, Montclair, NJ USA; 3 Department of Biostatistics and Health Data Science, School of Medicine, Indiana University, Indianapolis, IN USA; 4 Regenstrief Institute, Indiana University, Indianapolis, IN USA; 5 Indiana University Health, Indianapolis, IN USA

**Keywords:** Health services, Information systems and information technology

## Abstract

Medical coding is essential for healthcare operations yet remains predominantly manual, error-prone (up to 20%), and costly (up to $18.2 billion annually). Although large language models (LLMs) have shown promise in natural language processing, their application to medical coding has produced limited accuracy. In this study, we evaluated whether fine-tuning LLMs with specialized ICD-10 knowledge can automate code generation across clinical documentation. We adopted a two-phase approach: initial fine-tuning using 74,260 ICD-10 code–description pairs, followed by enhanced training to address linguistic and lexical variations. Evaluations using a proprietary model (GPT-4o mini) on a cloud platform and an open-source model (Llama) on local GPUs demonstrated that initial fine-tuning increased exact matching from <1% to 97%, while enhanced fine-tuning further improved performance in complex scenarios, with real-world clinical notes achieving 69.20% exact match and 87.16% category match. These findings indicate that domain-specific fine-tuned LLMs can reduce manual burdens and improve reliability.

## Introduction

Medical coding is a critical component of healthcare operations, involving the translation of clinical documentation into standardized codes essential for patient care, billing, research, and public health reporting^1–3^. Multiple coding systems exist—each with a unique design, hierarchical structure, and purpose. For instance, the International Classification of Diseases (ICD) is widely used globally for coding diseases and morbidity, encompassing over 74,260 unique codes in the ICD 10^th^ revision (ICD-10)^1,4^. In current practice, physicians often assign preliminary codes during patient visits and medical coders subsequently verify or correct these codes by reviewing the entire chart including unstructured clinical notes. Despite technological advancements, medical coding remains predominantly manual^4–6^, challenged by the immense variability in documentation styles and the sheer breadth of possible codes^5,7,8^. Consequently, this labor-intensive process is prone to human errors, reported to be as high as 20%^2,9,10^, contributing to inefficiencies within the substantial medical coding market, which totals approximately $18.2 billion annually in the US^11^.

Many prominent efforts have been made to enhance this process. Early efforts in the use of rule-based systems^12–15^ and terminology services like the Unified Medical Language System (UMLS) to map clinical terms to codes^16^. Researchers advanced to employing Natural Language Processing (NLP) techniques and machine learning approaches^17–21^ to automate medical coding tasks^5^. Notably, the Pretrained Language Models for ICD coding achieved a 59.8% match rate on ICD coding tasks involving approximately 8,900 codes, while the Label-Attention-Aware Transformer model reached 71.5% on the top 50 most frequently used codes^22,23^.

Despite these advancements, existing methods face significant limitations. They struggle with accurate named entity recognition (NER), the process of recognizing and extracting key medical terms for coding from clinical documentation^5,24,25^. This task is complicated by linguistic and lexical variations^5,26,27^, including informal shorthand (e.g., “HTN” for hypertension, “DM2” for type 2 diabetes), typographical errors (e.g., “malignnt” for malignant), and varied phrasing (e.g., “diabetic nephropathy” vs. “kidney disease due to diabetes”). Multiple interrelated conditions within a single note further complicate scaling to the full ICD-10 code set (e.g., “Type 2 diabetes with peripheral angiopathy with gangrene” requires both E11.52 and I70.26 codes). Traditional NLP approaches struggle with these complexities, particularly for context-driven reasoning^5^. Meanwhile, large language models (LLMs) have demonstrated strong natural language understanding and reasoning abilities^28–30^, offering promising potential^31^ to transform medical coding by interpreting complex medical contexts and generating accurate medical codes^32^.

Recent findings, however, indicate that LLMs have yet to meet the anticipated performance in medical coding, with accuracy below 50%^33^. Key challenges include: (1) generating non-existent codes, (2) struggling with less frequently used codes, and (3) assigning the same code to different clinical contexts, even when subtle distinctions warrant different codes. One approach uses prompt engineering to guide LLMs to select from a predefined subset of codes, but token limits prevent incorporating the entire ICD-10 set^34^. Another strategy employs Retrieval-Augmented Generation (RAG) to dynamically retrieve candidate codes, although the variability and errors in clinical documentation complicate accurate retrieval^35,36^.

Previous studies have predominantly focused on evaluating the parametric coding knowledge of LLMs using standardized code descriptions^33^, which do not sufficiently reflect the complexity of real-world clinical documentation. To address this limitation and the above challenges comprehensively, our study systematically evaluates four common linguistic and lexical variations encountered in clinical practice that affect coding accuracy^27,37–39^: (1) reordered diagnostic expressions, (2) typographical errors, (3) medical abbreviations, and (4) multiple interrelated conditions. Building on these, we further examine two more complex scenarios: (5) sentences with a single embedded diagnostic detail, and (6) full real-world clinical notes^33^. Our proposed methods target these variations to enhance the robustness and reliability of automated ICD-10 coding in clinical settings.

We hypothesize that LLMs’ limitations in medical coding stem from insufficient specialized knowledge. To test this hypothesis, we employed fine-tuning (training LLMs on domain-specific data to adapt them to a specific task^40^) using the complete ICD-10 code set and prompt engineering to avoid common errors. We experimented with both proprietary (e.g., OpenAI’s GPT-4o-mini^41^) and open-source LLMs (e.g., Meta’s Llama-3.2-1 B, Llama-3.2-3B, and Llama-3.1-8B^42^). This approach bypasses token limitations and enables LLMs to handle complex medical contexts. Rigorous evaluations using full real-world clinical notes evaluate the GPT of our approach in medical coding tasks and its potential to be adapted to other coding systems.

## Results

### Initial fine-tuning performance

We evaluated both pre-trained and fine-tuned models on standard ICD coding and linguistic and lexical variations. Fine-tuning dramatically improved performance, with the exact code-matching rate increasing from 3.35% to 97.48% for GPT-4o mini, and from 0.01–0.85% to 98.80–98.83% for Llama models in the base scenario. Among pre-trained models, GPT-4o mini (3.43–3.6%) outperformed Llama models (0.01–0.85%) across all tasks.

The model size was particularly influential in managing linguistic and lexical variations. In the Llama series, larger models consistently performed better after fine-tuning. For instance, in reordered descriptions, the exact code-matching rate rose from 36.19% (Llama-3.2-1B) to 78.23% (Llama-3.2-8B). A similar pattern was observed for sentence integration tasks, improving from 49.48% to 87.99%. All fine-tuned models handled medical abbreviations (92.86–95.27%) and typographical errors (58.43–83.84%) relatively well but struggled with multiple concurrent conditions (3.85–10.90%) and full real-world clinical notes (0.01%), indicating these remain challenging.

### Enhanced Fine-tuning Performance

Enhanced fine-tuning on GPT-4o mini and Llama-3.2-1B yielded substantial improvements across all variations (Table 1). For multiple concurrent conditions, Llama-3.2-1B went from 3.85% to 98.04%, and GPT-4o mini from 10.9% to 94.07%. Both models achieved an exact code-matching rate of 96.59% and 95.57% for medical abbreviations, 94.18% and 93.98% for typographical errors, and 97.32% and 93.92% for sentences with single embedded diagnostic elements in Llama and GPT-4o mini, respectively.

**Table 1:** Performance comparison of LLMs across pre-training, initial fine-tuning, and enhanced fine-tuning stages (*n* = 10,000)^a^

Model Type	Enhance Fine-tuned Models		Initial Fine-tuned Models				Pre-train Models			
Model	Llama-3.2-1B	GPT-4o mini	Llama-3.2-1B	Llama-3.2-3B	Llama-3.1-8B	GPT-4o mini	Llama-3.2-1B	Llama-3.2-3B	Llama-3.1-8B	GPT-4o mini
Reordered Diagnostic Expressions	87.51	85.02	36.19	67.92	78.23	61.30	0.01	0.13	0.69	3.31
Typographical Errors	94.18	93.98	58.43	80.31	69.10	83.84	0.01	0.07	0.79	3.58
Medical Abbreviations	96.59	95.57	93.80	92.86	95.27	93.06	0.01	0.06	0.07	3.55
Multiple Concurrent Conditions	98.04	94.07	3.85	4.18	5.24	10.90	0.00	0.06	0.27	3.43
Sentences with Single Embedded Diagnostic Information	97.32	93.92	49.48	76.85	87.99	64.61	0.00	0.10	0.85	3.60
Full Real-world Clinical Notes	69.20^c^	NA^b^	0.00	0.01	0.01	NA^b^	0.00	0.10	2.36	NA^b^

^a^All values represent the exact code-matching rate in percentages (%). Each model was evaluated on a test set of *n* = 10,000 samples.

^b^NA indicates data not available due to PhysioNet licensing restrictions, which explicitly prohibit sharing access to MIMIC data with external systems, such as GPT-4o. According to the license terms, “The LICENSEE will not share access to PhysioNet restricted data with anyone else”^48^.

^c^Represents Top-1 exact code-matching rate.

For the full real-world clinical notes, exact ICD code matching showed promising results: Top-1 accuracy reached 69.20% (95% CI: 67.42–71.09%) and Top-4 accuracy was 64.27% (95% CI: 63.25–8%). At the category level, the model demonstrated stronger performance with 87.16% (95% CI: 85.70-88.49%) Top-1 matching rate and 75.81% (95% CI: 74.90–76.71%) Top-4 matching rate. The MRR was 0.753, indicating an effective ranking of predictions. Table 2 presents detailed performance metrics across different ranking positions. All prediction accuracies were significantly better than random prediction (*p* < 0.001).

**Table 2:** Performance of enhanced fine-tuned Llama-3.2-1B on 10,000 full real-world clinical notes by ICD-10 code

Test Codes	Total Codes	Exact Matched Codes (Rate)	Exact Match 95% CI^a^ | *P*-value^e^	Categorical Matched Codes (Rate)	Categorical Match 95% CI^a^ | *P*-value^e^
Top-1^b^	2211	1530 (69.2%)	[67.42, 71.09] | *p* < 0.001	1,927 (87.16%)	[85.70, 88.49] | *p* < 0.001
Top-2	4410	2860 (64.85)	[63.43, 66.25] | *p* < 0.001	3,484 (79.02%)	[77.79, 80.20] | *p* < 0.001
Top-3	6554	4219 (64.37)	[63.21, 65.52] | *p* < 0.001	5,042 (76.94%)	[75.91, 77.95] | *p* < 0.001
Top-4	8590	5521 (64.27)	[63.25, 65.28] | *p* < 0.001	6,510 (75.81%)	[74.90, 76.71] | *p* < 0.001

^a^CI indicates confidence interval, calculated using the Wilson score interval method.

^b^Top-1 indicates the model’s first prediction matches the ground truth.

^c^Exact Code Match requires complete ICD-10 code matching.

^d^Category Match only requires matching of the first three characters of the ICD-10 code.

^e^The *P*-value indicates the statistical significance of the accuracy being better than expected random. A smaller *p*-value suggests stronger evidence of non-random prediction performance.

### Error Analysis

**Fig. 1:**
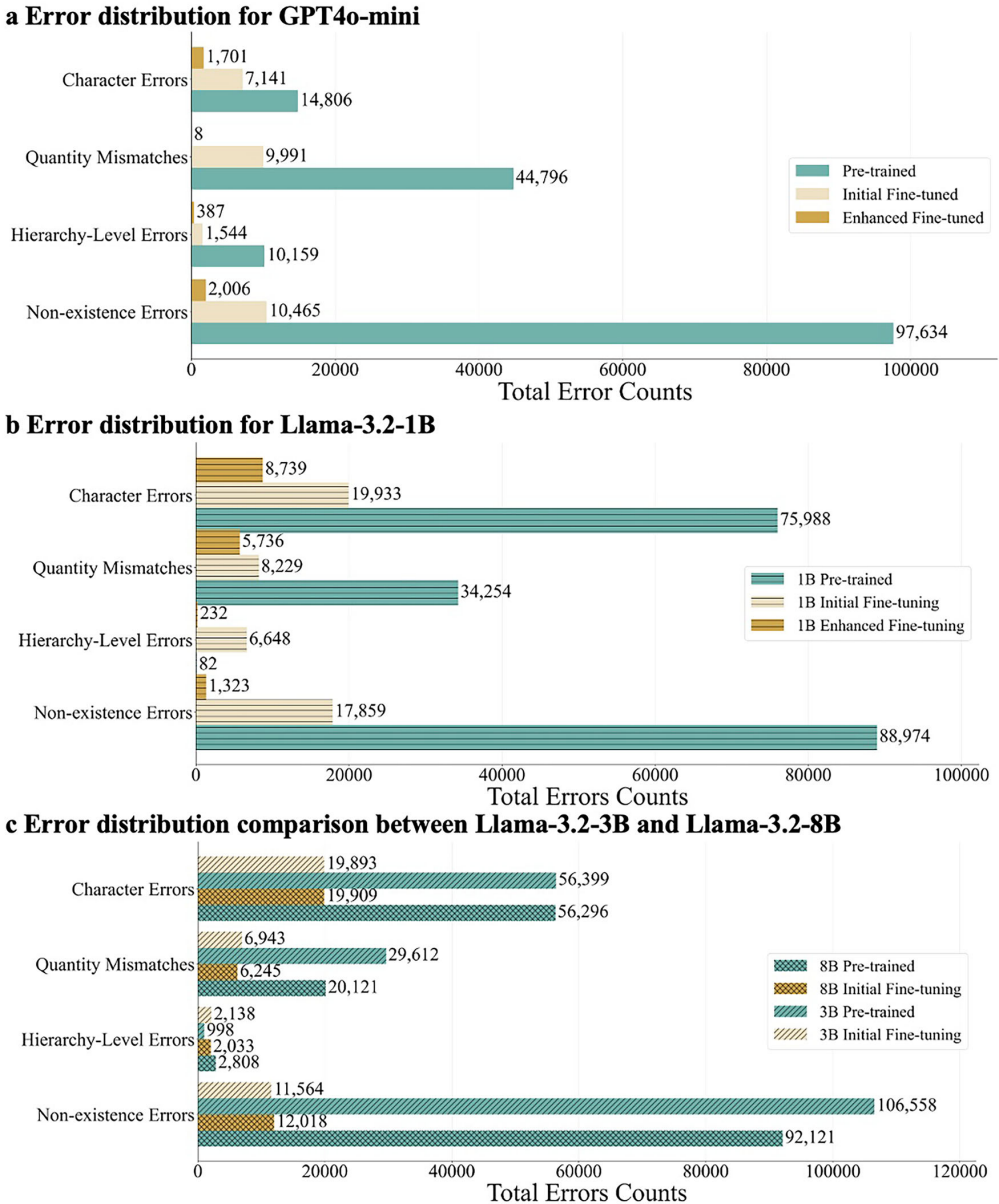
Error distribution pattern comparison across model scales and training stages. Part **a** details errors for GPT-4o mini, Part **b** for Llama-3.2-1B, and Part **c** compares Llama-3.2-3B and Llama-3.2-8B. Non-existence errors potentially cause claim rejections and billing failures in healthcare systems. Hierarchy-level errors affect reimbursement amounts and clinical decision support by missing diagnostic specificity. Quantity mismatches fail to capture critical comorbidities that influence treatment plans. Character errors involve transposed digits or typographical mistakes in codes that may lead to incorrect billing or documentation, affecting data integrity. The higher error counts in Llama models are due to the inclusion of 10,000 additional MIMIC test samples in their evaluation. All models demonstrate similar error reduction trends, with enhanced fine-tuning delivering further improvements across all error categories.

Furthermore, we performed an error analysis focusing on the recognized most frequently occurring errors, categorized into four types: (1) *Non-existence Errors* as generating invalid ICD codes; (2) *Hierarchy-Level Errors* related to incorrect specificity in the ICD hierarchy; (3) *Quantity Mismatches*, which means generating an incorrect number of codes; and (4) *Character Errors* such as typos or transposed digits^33^. As shown in Fig. 1, most error types decreased significantly after fine-tuning compared to the pre-trained models, although hierarchy-level errors increased in some instances due to attempts at more specific coding rather than defaulting to broader categories. This trend was consistent across all model variants, underscoring the effectiveness of our approach.

In the Llama series (Fig. 1b and c), non-existence errors generally declined as model size increased from 1B to 8B, though the 3B model showed a small deviation. In contrast, differences in quantity mismatches and character errors were less scale dependent. GPT-4o mini followed similar error patterns (Fig. 1a), with all error types decreasing further after enhanced fine-tuning. The consistency across different LLMs suggests the robustness of our training approach in reducing various error types^43^.

For full real-world clinical notes, analysis of 535 incorrect codes from 200 random samples revealed four distinct error patterns: (1) *Information Absence* (42%), where no relevant documentation supported the assigned code (e.g., a tobacco dependence code with no mention of smoking); (2) *Diagnostic Criteria Insufficiency* (38%), where basic symptoms a were present but lack specific metrics needed for definitive diagnosis (e.g., mentioning renal insufficiency without echocardiography confirmation for mitral insufficiency staging)^44^; (3) *Clinical Context Misinterpretation* (8%) where the model fails to properly understand clinical context, such as in a sample where “history of cervical cancer s/p hysterectomy with no evidence of recurrence” was incorrectly coded as current cervical cancer; and (4) *Coding Rule Violation* (12%) where ICD guidelines were incorrectly applied, such as using separate codes for hypertension and chronic kidney disease instead of the combination code (I12.9).

## Discussion

We hypothesized that the limitations of LLMs in medical coding stem primarily from insufficient domain-specific coding knowledge rather than inherent model deficiencies. Our experimental results support this hypothesis through a two-stage validation process. Initial fine-tuning with the complete ICD-10 dataset improved the exact code-matching rate from less than 1% to over 97% in the base scenario. Enhanced fine-tuning further enabled the models to manage various documentation challenges, achieving high performance on reordered diagnostic expressions (97.32%), medical abbreviations (95.57%), and typographical errors (94.18%). For full real-world clinical notes, our enhanced fine-tuned model reached 69.20% on exact code matching and 88.85% at the category level.

Error analysis revealed distinct patterns across different evaluation scenarios. In the base scenario, most error types decreased after fine-tuning, although hierarchy-level errors rose slightly as models attempted more precise coding. Balanced hierarchical training may help refine the matching accuracy at the granular code level. In full clinical notes, challenges emerged in synthesizing scattered clinical details (chief complaints, present illness, physical examination, etc.) and performing complex clinical reasoning. This limitation could be addressed by integrating additional external information such as lab results and imaging reports. Clinical reasoning errors manifested in difficulties with implicit information understanding, temporal logic processing, multi-condition synthesis, and uncertainty interpretation. For instance, the models sometimes failed to infer that “stable vital signs” in a postoperative patient indicate good recovery or that “mild elevation of blood pressure” in a pregnant woman could suggest preeclampsia risk. Furthermore, they also struggled with temporal relationships (history, current episode, treatment process), accurately differentiating symptoms from various periods, and handling multi-condition logic. Additionally, doctors’ uncertainty phrases (e.g., “consider,” “to be excluded”) were often misinterpreted. Addressing these deeper reasoning challenges will require structured reasoning templates, enhanced training data capturing complex temporal and conditional relationships, standardized outputs (e.g., confidence levels), and layered evaluation mechanisms.

Despite the promising results, the approach has limitations, including the substantial computational resources and time required for fine-tuning, as well as the need for extensive training data to achieve optimal performance. Although the models perform well on many tasks, manual review by domain experts remains essential, particularly for complex or nuanced cases. Importantly, our approach is intended to support human coders, reduce manual burdens, and allow coders to focus on verification rather than exhaustive coding tasks. Another limitation of our study is that, although we now include a significant portion of less frequently assigned ICD-10 codes appearing in discharge summaries, explicit evaluation on truly rare codes remains unexplored.

Future work will aim to address these limitations and broaden the applicability of our approach. We plan to evaluate the proposed approach on other LLMs, develop advanced prompt engineering techniques, and evaluate these systems in real-world clinical environments. We also plan to further investigate the capabilities of generative LLMs in handling genuinely rare codes, leveraging their contextual reasoning advantages over traditional supervised methods that require substantial training examples. This direction could further enhance the generalizability and clinical applicability of automated medical coding systems. Additionally, exploring interoperability across healthcare standards, such as Fast Healthcare Interoperability Resources (FHIR)^45^ and Observational Medical Outcomes Partnership (OMOP)^46,47^, to automate data translation and integration. By enabling LLMs to seamlessly handle diverse healthcare data formats, this approach has the potential to transform how information flows across the healthcare ecosystem.

In this study, we introduced a novel two-stage fine-tuning approach that enables LLMs to effectively automate medical coding tasks across diverse real-world clinical documentation scenarios. Compared to existing works using LLMs for medical coding, which primarily evaluate models based on parametric knowledge rather than real-world clinical complexity, our method provides a more comprehensive framework that systematically addresses realistic medical coding challenges frequently encountered in clinical practice. Our findings validate our hypothesis that specialized medical coding knowledge can be effectively imparted to LLMs through fine-tuning, and overcoming challenges such as token limitations, reliance on pre-selected code sets, and character-level hallucination. Moreover, our approach highlights the capability of fine-tuned LLMs to accurately map clinical entities to standardized codes, aligning closely with the goals of interoperable electronic health record systems. Moreover, our methodology lays a robust foundation for extending these techniques to other coding systems and diverse healthcare datasets, thereby supporting more precise, efficient, and interoperable healthcare data management.

## Method

**Fig. 2:**
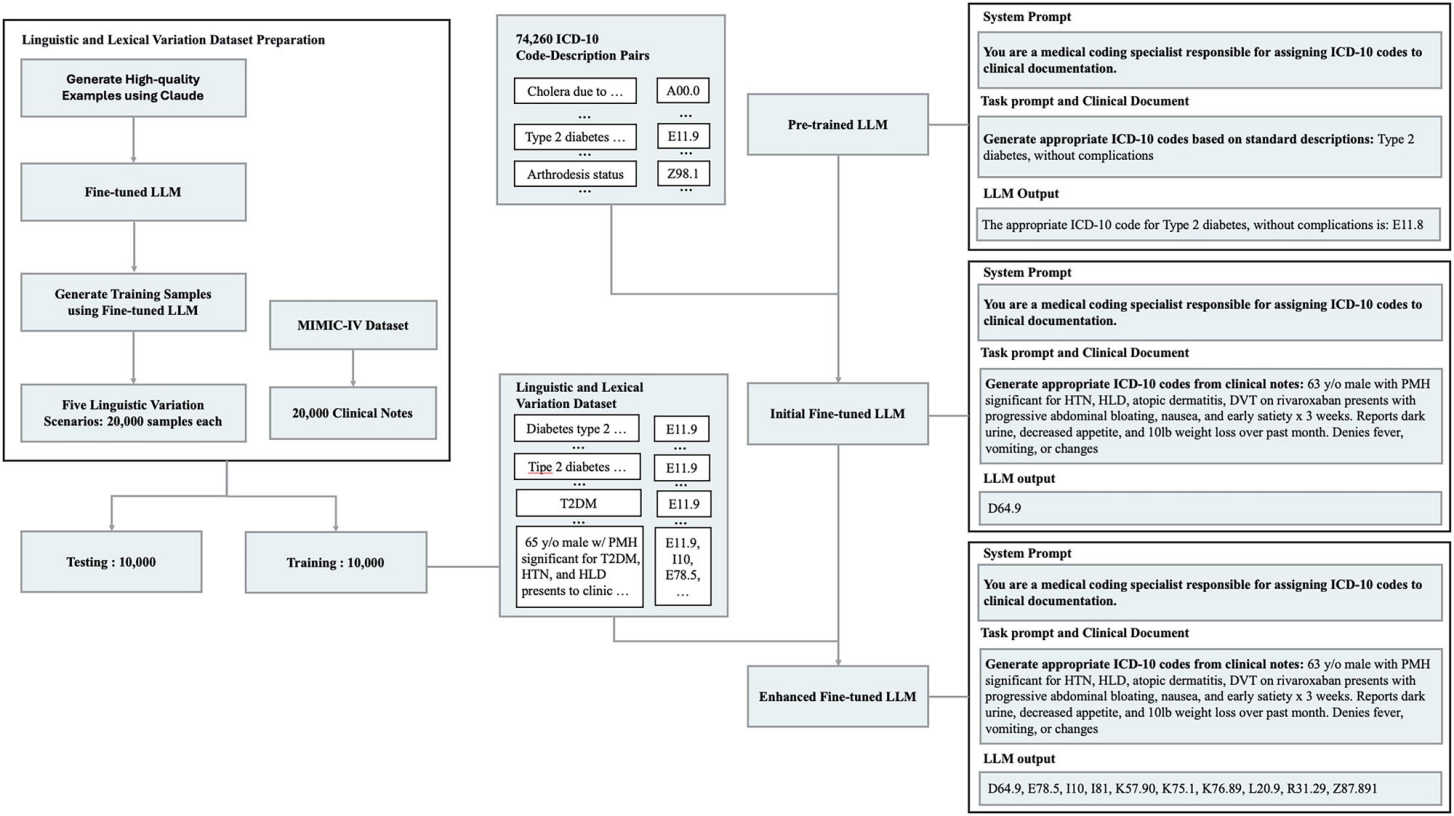
Two-stage fine-tuning framework initial knowledge integration and enhanced clinical variation processing. This figure outlines our fine-tuning process. The left side focuses on preparing data to handle variations in how clinical text is written. The middle section shows the training process, starting with learning from the full ICD-10 dataset and then fine-tuning further for specific clinical variations. The right side provides examples that illustrate how accuracy in coding improves across different medical scenarios.

To effectively impart medical coding knowledge to LLMs, we designed a two-step fine-tuning framework (Fig. 2). In the initial stage, we leveraged the complete ICD-10 code set comprising 74,260 code-description pairs across A–Z classifications and structured the training process as input-output pairs (e.g., pairing code “E11.9” with “Type 2 diabetes mellitus without complications”. The enhanced fine-tuning stage targets four coding challenges arising from linguistic and lexical variations in clinical notes. For each variation type, specialized training objectives and targeted data augmentation strategies were implemented.

### Data preparation

We prepared two datasets to be used in the initial fine-tuning and enhanced fine-tuning procedures. For the base scenario, we utilized the complete ICD-10-CM code-description pairs for training and testing, ensuring full coverage of all ICD-10 codes.

As illustrated in Fig. 2 (left), for linguistic and lexical variation scenarios (excluding clinical notes), we developed a pipeline to generate clinically relevant test data. First, for each variation type, we created 10 high-quality example pairs using Claude (e.g., “Generate ICD-10 descriptions that incorporate one or more standard medical abbreviations, such as converting ‘unspecified’ to ‘unspec.’, while maintaining word order and meaning”). These examples were then used directly as training samples to fine-tune the model (see Supplementary Table 1, Supplementary Table 2, Supplementary Table 3, Supplementary Table 4, and Supplementary Table 5 for detailed prompts)^48^. Using these curated examples as templates, we then generated 20,000 samples via Application Programming Interface (API) calls, preserving clinical authenticity and coding complexity (see Supplementary Table 6 for detailed prompts and generation methodology); the samples were equally split into 10,000 each for training and testing, For each variation scenario, we used completely distinct ICD-10 code sets between the training and testing datasets to robustly assess generalization performance.

The data generated encompasses several variations:

1. Reordered Diagnostic Expressions: Rearranging word order in standard ICD-10 descriptions while preserving semantic meaning, to evaluate the model’s adaptability to varied phrasing.
2. Typographical Errors: Injecting spelling errors based on text length^38^ (shorter descriptions <10 words receive 1–2 errors, while longer ones >20 words may receive up to 4 errors^39^), to assess robustness to misspellings.
3. Medical Abbreviations: Replacing standard medical terms with common abbreviations (e.g., “unspecified” to “unspec.”), testing the model’s capacity to interpret frequently used clinical shorthand.
4. Multiple Concurrent Conditions: Combining two to five ICD-10 descriptions to reflect comorbidities to evaluate the model’s ability to process comorbidities.
5. Sentences with Single Embedded Diagnostic Information: Embedding ICD-10 descriptions into approximately 23 words, a length sufficient to effectively represent a complete diagnostic context while maintaining efficiency^49^, to test the model’s ability to extract relevant entities and generate corresponding codes.

Table 3 presents examples of each test type along with their corresponding standard codes, which aim to comprehensively evaluate the model’s capability to handle diverse and challenging clinical scenarios^10,50–52^. For the full real-world clinical notes coding scenario, we utilized discharge summaries from the Medical Information Mart for Intensive Care (MIMIC)-IV dataset—a large, publicly available database containing de-identified electronic health records from intensive care units at the Beth Israel Deaconess Medical Center in Boston. Due to MIMIC data use restrictions prohibiting data sharing with external services such as ChatGPT, this evaluation used only open-source Llama models in a local setting^48^. To assess the accuracy of our coding approach, we concentrated on the top four ICD codes ordered by their priority in each discharge summary, as these codes typically represent the most critical diagnoses for each admission documented by MIMIC^53^. To ensure robustness and avoid overfitting, we randomly selected 10,000 discharge summaries from the MIMIC-IV dataset for training. For evaluation, we specifically tested on 2211 discharge summaries from a separate set of 10,000 randomly selected records, restricting our test cases to admissions primarily related to circulatory system diseases, given their high prevalence and clinical significance in intensive care settings^54^. We performed statistical checks to compare the training and testing sets. At the diagnostic category level (first three ICD characters), chi-square tests revealed significant differences (χ² = 985.5, *p* < 0.001). Low-frequency codes (occurring in <0.1% of records) constitute approximately 34.60% of unique codes, ensuring evaluation across both common and uncommon diagnostic patterns. A detailed code distribution across training and testing datasets are provided in the Supplementary Fig. 1 and Supplementary Note 1. These results confirm that the training and testing sets capture distinct real-world variability in diagnostic patterns, supporting the generalizability of our work.

**Table 3:** Test scenario examples with standard ICD-10 codes

Test Type	Standard Description	Example Description	Code
Base	Type 2 diabetes mellitus without complications	Type 2 diabetes mellitus without complications	E119
Reordered Diagnostic Expressions	Burkitt lymphoma, intrathoracic lymph nodes	Intrathoracic lymph nodes Burkitt lymphoma	C8372
Typographical Errors	Foreign body granuloma of soft tissue, not elsewhere classified, unspecified hand	Foriegn body granuloma of soft tisue, not classifed elsewhere, unspecifid hand	M6024
Medical Abbreviations	Acute respiratory failure, unspecified whether with hypoxia or hypercapnia	Acute resp. failure, unspec. whether w/ hypoxia or hypercap.	J9600
Multiple Concurrent Conditions	External constriction of vagina and vulva, initial encounter. Phocomelia, unspecified limb(s)	External constriction of vagina and vulva, initial encounter. Phocomelia, unspecified limb(s)	S30844A, Q731
Sentences with Single Embedded Diagnostic Information	Terrorism involving firearms, public safety official injured, sequela	Public safety official, Mike, was injured as a result of terrorism involving firearms, and he has sequela related to that incident.	Y384X1S
Full Real-world Clinical Notes	Anemia, unspecified. Hyperlipidemia, unspecified. Essential (primary) hypertension. Portal vein thrombosis. Diverticulosis of intestine, part unspecified, without perforation or abscess without bleeding. Phlebitis of portal vein. Other specified diseases of the liver. Atopic dermatitis, unspecified. Other microscopic hematuria. Personal history of nicotine dependence. Presence of intraocular lens.	___ man with HTN, HLD, atopic dermatitis [omitted other past medical history - 82 words] presenting with abdominal bloating and nausea for several weeks. CT showed portal vein thrombi [omitted detailed imaging findings - 45 words]. [omitted detailed lab results - 156 words] Diagnosed with pylephlebitis, treated with anticoagulation (rivaroxaban) and antibiotics (Cipro/Flagyl) [omitted specific dosing - 38 words]. [omitted detailed physical exams - 112 words] Discharged home with 4-week antibiotic course and follow-up plan [omitted detailed follow-up instructions - 218 words].	I10, D649, I81, E785, K5790, K751, K7689, L209, R3129, Z87891, Z961

### Experiment setup

This study evaluated both open-source and proprietary LLMs to examine how different architectures and model sizes respond to fine-tuning for medical coding tasks. From the open-source Llama series, we selected Llama3 models with one billion, three billion, and eight billion parameters to investigate the impact of model size. For proprietary models, we employed the GPT-4o mini (released July 2024), which is based on the latest GPT-4 architecture. Although GPT-4 demonstrated superior performance, its restricted API led us to choose GPT-4o mini for its sufficient performance^41^. For enhanced fine-tuning, we selected GPT-4o mini and Llama-3.2-1B; the latter’s minimal memory footprint (2GB GPU), is ideal for local deployment in resource-constrained environments, while GPT-4o mini serves as our cloud-based option, balancing performance and computational cost^55,56^.

Experiments were conducted in two environments: GPT-4o mini was fine-tuned on OpenAI’s cloud platform with a learning rate multiplier of 1.8, while Llama models were locally fine-tuned on four NVIDIA A100 GPUs (80GB each) using Llama Factory^57^ with DeepSpeed^58^ optimization. Following Soroush et al.‘s findings that ICD-10 descriptions can be effectively processed with limited token length, we used full parameter fine-tuning with a maximum sequence length of 50 tokens^33^. The number of epochs was set to 10, and the base batch size was set to one per device with dynamic adjustment based on memory utilization^59^. We employed the AdamW optimizer with a learning rate of 1e^-5^ and a cosine learning rate scheduler^60^.

### Evaluation prompting and metrics

We adopted a two-stage prompt structure (Fig. 2). The system prompt sets the model’s role: “You are a medical coding specialist responsible for assigning ICD-10 codes to clinical documentation”, and the input prompt specifies the task type (“Generate appropriate ICD-10 codes from clinical notes” or “Generate appropriate ICD-10 codes based on standard descriptions”), depending on the scenario.

We rigorously assessed performance with exact code matching, requiring correct identification of both the medical entity (NER match) and its corresponding ICD-10 code (code match) and category matching with the category code level (e.g., “E11” for diabetes) to determine whether the model identified the correct disease category. We calculated the exact match accuracy to statistically analyze model performance. The *P*-value indicates the statistical significance of the accuracy being better than random prediction. We additionally employed the Top‑N accuracy (with N ranging from one to four based on prediction rank priority^53^) and Mean Reciprocal Rank (MRR) in full real‑world clinical note scenarios, where medical codes are assigned in priority order. For each metric, we present both the accuracy percentage and its 95% confidence interval (CI). The Top-N metric reflects the percentage of instances in which the correct code appears within the top N predicted codes, while MRR helps evaluate the overall ranking quality of our predictions.

## Supplementary information

Supplementary Tables

## Data Availability

Data from this study were derived from the MIMIC-IV discharge summaries, which are available upon approved request through PhysioNet https://physionet.org/content/mimiciv/. Our implementation code, data analysis scripts, and data index used for training and testing are available at https://github.com/hzvictor/LLMCoder.

## Code availability

Our implementation code and data analysis scripts used for training and testing are available at https://github.com/hzvictor/LLMCoder.
