# Supplementary material for: Enhancing medical coding efficiency through domain-specific fine-tuned large language models
Source: Npj Health Syst. 2025 May 1;2:14. doi: 10.1038/s44401-025-00018-3 (PMC12045799; doi:10.1038/s44401-025-00018-3)
Supplement: Supplementary file 1 — Supplementary Tables [file 44401_2025_18_MOESM1_ESM.pdf]

Enhancing Medical Coding Efficiency through Domain-Specific Fine-Tuned Large Language Models

The content of this supplement is:

1. **Supplementary Table 1.** Prompt and Examples of Reordered Diagnostic Expressions.
2. **Supplementary Table 2.** Prompt and Examples of Typographical Errors.
3. **Supplementary Table 3.** Prompt and Examples of Medical Abbreviations.
4. **Supplementary Table 4.** Approach and Examples of Multiple Concurrent Conditions Combined Descriptions.
5. **Supplementary Table 5.** Examples of Sentences with Single Embedded Diagnostic Information
6. **Supplementary Table 6.** Prompts Used for Fine-tuning Models by Variation Type
7. **Supplementary Figure 1.** Scatter Plot Comparing ICD Category Frequencies Between Training and Testing Sets
8. **Supplementary Note 1.** Low-Frequency Code Analysis in Testing Dataset

1. Supplementary Table 1. Prompt and Examples of Reordered Diagnostic Expressions.

Prompt	Standard Description	Reordered Description	ICD-10 Code
For each ICD-10 description provided, please create a reordered version that maintains the same clinical meaning. The reordering should follow natural language patterns that might appear in clinical documentation while ensuring the clinical meaning remains unchanged.<br>Example Interactive Pair Generation:<br>Input: "Type 2 diabetes mellitus without complications"<br>Output: "Diabetes mellitus type 2 without complications"	Viral intestinal infection, unspecified	Unspecified intestinal viral infection	A084
	Cholera due to Vibrio cholerae 01, biovar eltor	Biovar eltor cholera caused by Vibrio cholerae 01	A001
	Enterocolitis due to Clostridium difficile, not specified as recurrent	Clostridium difficile enterocolitis, not specified as recurrent	A047
	Botulism food poisoning	Food poisoning by botulism	A051
	Foodborne Clostridium perfringens [Clostridium welchii] intoxication	Clostridium perfringens [Clostridium welchii] foodborne intoxication	A052
	Tuberculosis of digestive tract organs, not elsewhere classified	Digestive tract organs tuberculosis, not classified elsewhere	A183
	Cutaneous anthrax	Anthrax, cutaneous	A220
	Disseminated mycobacterium avium-intracellulare complex (DMAC)	Mycobacterium avium-intracellulare complex, disseminated (DMAC)	A318
	2-part displaced fracture of surgical neck of unspecified humerus, subsequent encounter for fracture with routine healing	Subsequent encounter for 2-part displaced fracture of surgical neck of unspecified humerus with routine healing	S42202D
	2-part displaced fracture of surgical neck of unspecified humerus, subsequent encounter for fracture with delayed healing	Subsequent encounter for 2-part displaced fracture of surgical neck of unspecified humerus with delayed healing	S42202G

2. Supplementary Table 2. Prompt and Examples of Typographical Errors.

Prompt	Standard Description	Description with Errors	ICD-10 Code
For each ICD-10 description I provide, create a version with realistic typographical errors following these guidelines:<br>- 1-2 errors for descriptions <10 words<br>- 2-3 errors for descriptions 10-20 words<br>- 3-4 errors for descriptions >20 words<br>Errors should reflect common typing mistakes in clinical documentation, such as transposed letters, missing letters, extra letters, and similar-looking letter errors.<br>Example Interactive Pair Generation:<br>Input: "Type 2 diabetes mellitus without complications" Output: "Tyep 2 diabets mellitus witout complications"	Foreign body granuloma of soft tissue, not elsewhere classified, unspecified hand	Foriegn body granuloma of soft tisue, not classifed elsewhere, unspecifid hand	M60842
	Disease of spinal cord, unspecified	Desease of spinl cord, unspecifid	G959
	Acute respiratory failure, unspecified whether with hypoxia or hypercapnia	Acute respiratry failur, unspecifid whether with hypoxia or hypercapnia	J9600
	Hordeolum internum right eye, unspecified eyelid	Hordeolm internum rihgt eye, unspecifid eyelid	H00015
	Asphyxiation due to hanging, undetermined, subsequent encounter	Asphyxiaton due to hangin, undetermind, subsequnt encounter	T71163D
	Pressure-induced deep tissue damage of sacral region	Presure-inducd deep tisue damage of sacral region	L89159
	Skeletal fluorosis, right shoulder	Skeltal fluorosis, rihgt shouldr	M85811
	Kaposi's sarcoma, unspecified	Kaposis sarcoma, unspecifid	C469
	Prostatic intraepithelial neoplasia	Prostatic intraepithelil neoplasia	N423
	Displaced spiral fracture of shaft of radius, left arm, subsequent encounter for open fracture type IIIA, IIIB, or IIIC with routine healing	Displeced spiral fractur of shaft of radus, left arm, subsequnt encountr for open fractur type IIIA, IIIB, or IIIC with routin healing	S52392A

3. Supplementary Table 3. Prompt and Examples of Medical Abbreviations.

Prompt	Standard Description	Abbreviated Description	ICD-10 Code	Abbreviations Used
For each ICD-10 description I provide, create a version using standard medical abbreviations commonly found in clinical documentation. Use appropriate clinical abbreviations (such as "w/" for "with", "unspec." for "unspecified", "resp." for "respiratory", etc.) while maintaining clinical clarity.<br><br>Example Interactive Pair Generation:<br><br>Input: "Type 2 diabetes mellitus without complications"<br><br>Output: "Type 2 DM w/o complications"	Foreign body granuloma of soft tissue, not elsewhere classified, unspecified hand	Foreign body gran. of soft tissue, not elsewhere class., unspec. hand	M60842	gran.: granuloma, class.: classified, unspec.: unspecified
	Disease of spinal cord, unspecified	Disease of spin. cord, unspec.	G959	spin.: spinal, unspec.: unspecified
	Acute respiratory failure, unspecified whether with hypoxia or hypercapnia	Acute resp. failure, unspec. whether w/ hypoxia or hypercap.	J9600	resp.: respiratory, unspec.: unspecified, w/: with, hypercap.: hypercapnia
	Hordeolum internum right eye, unspecified eyelid	Hordeolum int. rt. eye, unspec. eyelid	H00015	int.: internum, rt.: right, unspec.: unspecified
	Asphyxiation due to hanging, undetermined, subsequent encounter	Asphyx. due to hanging, undet., subseq. enc.	T71163D	Asphyx.: Asphyxiation, undet.: undetermined, subseq.: subsequent, enc.: encounter
	Pressure-induced deep tissue damage of sacral region	Press.-induced deep tiss. damage of sacr. region	L89154	Press.: Pressure, tiss.: tissue, sacr.: sacral
	Skeletal fluorosis, right shoulder	Skel. fluorosis, rt. shoulder	M85811	Skel.: Skeletal, rt.: right
	Kaposi's sarcoma, unspecified	Kaposi's sarc., unspec.	C469	sarc.: sarcoma, unspec.: unspecified
	Prostatic intraepithelial neoplasia	Prostatic intraepith. neoplasia	N423	intraepith.: intraepithelial, neoplasia: neoplasia
	Cardiac murmur, unspecified	Card. murmur, unspec.	R011	Card.: Cardiac, unspec.: unspecified

4. Supplementary Table 4. Approach and Examples of Multiple Concurrent Conditions Combined Descriptions.

Approach	Combined Description	Individual Descriptions	ICD-10 Codes
A Python script is created to randomly combine two to five standard ICD-10 descriptions into a single text.	Injury of right internal carotid artery, intracranial portion, not elsewhere classified, sequela. Rupture of synovium, right ankle and foot, sequela. Burn of third degree of multiple sites of right wrist and hand, sequela. Adverse effect of unspecified drugs, medicaments and biological substances, initial encounter	1. Injury of right internal carotid artery, intracranial portion, not elsewhere classified, sequela<br>2. Rupture of synovium, right ankle and foot, sequela<br>3. Burn of third degree of multiple sites of right wrist and hand, sequela<br>4. Adverse effect of unspecified drugs, medicaments and biological substances, initial encounter	S0681A, S66171, S23391A, T5099
	Displaced fracture of triquetrum [cuneiform] bone of left wrist. Driver of pick-up truck or van injured in collision with car, pick-up truck or van in nontraffic accident, initial encounter	1. Displaced fracture of triquetrum [cuneiform] bone of left wrist<br>2. Driver of pick-up truck or van injured in collision with car, pick-up truck or van in nontraffic accident, initial encounter	S62112A, V530XXS

5. Supplementary Table 5. Prompt and Generated Reordered Diagnostic Examples.

Prompt	Clinical Sentence	Standard Description	ICD-10 Code
For each ICD-10 code and description provided, create a natural-sounding clinical sentence that embeds this diagnostic information within relevant context. The sentence should reflect realistic clinical documentation while preserving the key diagnostic elements.<br><br>Example Interactive Pair Generation:<br><br>Input: "Type 2 diabetes mellitus without complications"<br><br>Output: "John, a	Mary, a 45-year-old woman, suffers from an unspecified disease affecting her spinal cord, which is causing her significant discomfort and limiting her mobility.	Disease of spinal cord, unspecified	G959
	Tom, a 68-year-old man, recently experienced a severe episode of acute respiratory failure, and the medical team is currently unspecified whether it was accompanied by hypoxia or hypercapnia.	Acute respiratory failure, unspecified whether with hypoxia or hypercapnia	J9600
	Sarah, a 27-year-old patient, has developed a painful hordeolum internum in her right eye, affecting an unspecified eyelid, which is causing her discomfort and affecting her vision.	Hordeolum internum right eye, unspecified eyelid	H00023
	Mike, a 32-year-old male, had a subsequent medical encounter related to asphyxiation due to hanging, and the circumstances surrounding the incident remain undetermined.	Asphyxiation due to hanging, undetermined, subsequent encounter	T71164D
	Emily, a 78-year-old woman, has been diagnosed with pressure-induced deep tissue damage in her sacral region, which is causing her significant pain and discomfort.	Pressure-induced deep tissue damage of sacral region	L89156
	David, a 54-year-old man who works in an industrial setting, suffers from skeletal fluorosis affecting his right shoulder, which is causing him chronic pain and limited range of motion.	Skeletal fluorosis, right shoulder	M85111
	Lisa, a 42-year-old woman, has recently been diagnosed with Kaposi's sarcoma, a type of cancer, but the specific location of the tumor is currently unspecified.	Kaposi's sarcoma, unspecified	C469
	Robert, a 62-year-old man, has been diagnosed with prostatic intraepithelial neoplasia, a precancerous condition affecting his prostate gland.	Prostatic intraepithelial neoplasia	N4231

57-year-old man with a 10-year history of type 2 diabetes mellitus without complications, continues to maintain good glycemic control through diet, exercise, and oral medications."	William, a 39-year-old male, had a subsequent medical encounter for a displaced spiral fracture of the shaft of his left radius, which was an open fracture type IIIA, IIIB, or IIIC and is now healing routinely.	Displaced spiral fracture of shaft of radius, left arm, subsequent encounter for open fracture type IIIA, IIIB, or IIIC with routine healing	S52342F
	Emma, a 28-year-old female athlete, had a subsequent medical encounter for a nondisplaced transverse fracture of her left patella, which was an open fracture type IIIA, IIIB, or IIIC and has developed a nonunion.	Nondisplaced transverse fracture of left patella, subsequent encounter for open fracture type IIIA, IIIB, or IIIC with nonunion	S82035N

6. Supplementary Table 6. Prompts Used for Fine-tuning Models by Variation Type.

Prompt	Variation Type
Generate output sentences describing a person's condition based on input ICD-10 descriptions. Mention the person's name and specific condition.	Sentences with Single Embedded Diagnostic Information
Generate input ICD-10 descriptions using standard medical abbreviations, maintaining word order and medical meaning.	Medical Abbreviations
Generate input ICD-10 descriptions with typos (1-4 based on length), maintaining word order and medical meaning.	Typographical Errors
Reorder the input ICD description without changing the meaning.	Reordered Diagnostic Expressions

Using our 10 high-quality example pairs for each variation type, we created structured JSONL files containing system prompts, input descriptions, and transformed outputs, then uploaded these files to OpenAI's platform to fine-tune GPT-4o mini. For each variation category, we developed a specialized model capable of applying the particular linguistic or lexical transformation patterns. The fine-tuning process utilized a learning rate of 1.8×10^{-5}, 10 training epochs, and a maximum sequence length of 1000 tokens. Each model was trained specifically to transform standard ICD-10 descriptions into one particular variation while maintaining clinical accuracy. Detailed prompts used for each variation type are provided in the table. We generated 20,000 examples for each variation type by implementing a batched API calling strategy for efficient large-scale data generation. When API calls failed during the data generation process, our system automatically triggered regeneration attempts. For additional implementation details, please refer to our open-source repository at https://github.com/hzvictor/LLMCoder.

7. Supplementary Figure 1. Scatter Plot Comparing ICD Code Counts Between Training and Testing Sets

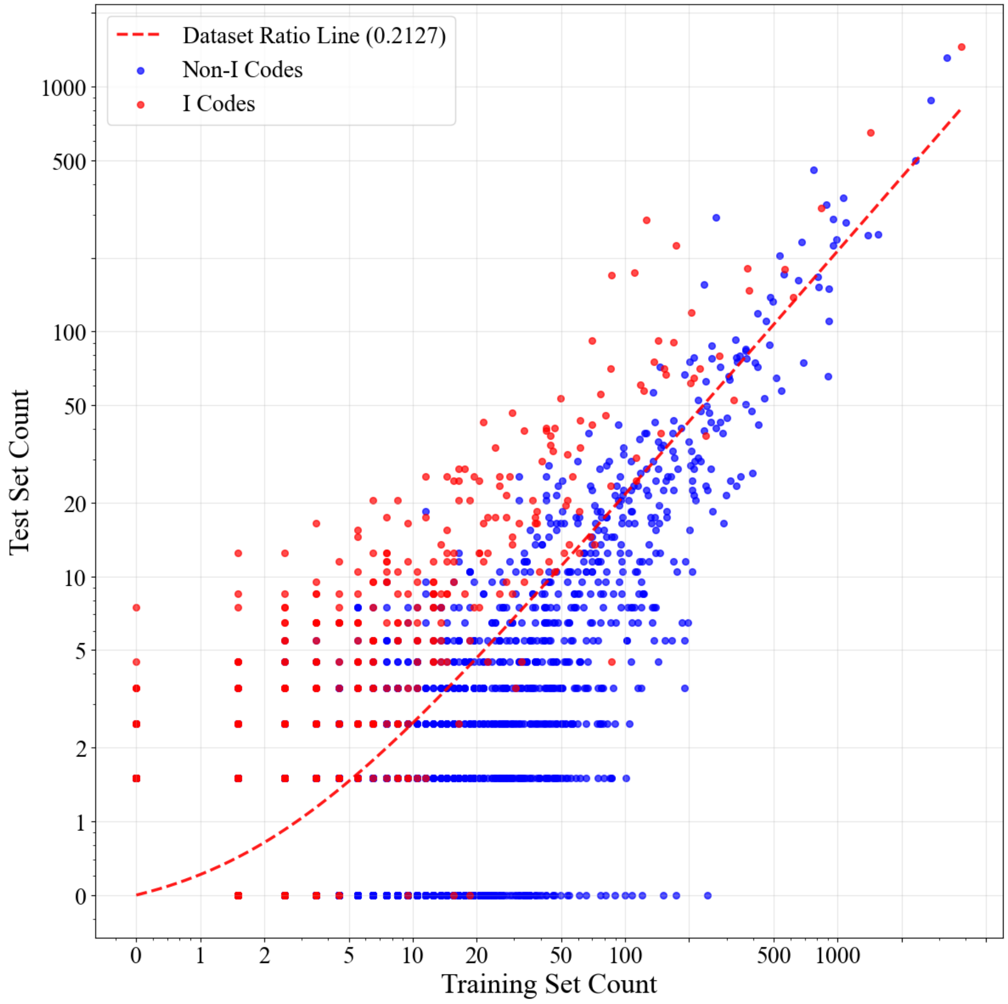

Supplementary Figure 1: Scatter plot comparing ICD code counts between training and testing sets. The horizontal axis represents count in the training set, the vertical axis shows count in the testing set, with blue points representing non-I codes and red points representing I codes (circulatory system diseases). An offset has been added to all counts to display zeros on the logarithmic scale. The dashed red line represents the expected distribution based on the dataset size ratio.

8. Supplementary Note 1. Low-Frequency Code Analysis in Testing Dataset

In this study, we defined "low-frequency codes" as ICD-10 codes with occurrence frequencies below 0.1% in the entire MIMIC-IV dataset. Our analysis of the testing dataset revealed that among the total 24,384 code occurrences, 8,438 (34.60%) were instances of low-frequency codes, comprising 1,902 unique low-frequency codes. When examining only the top 4 positions, we found that out of 11,626 code occurrences, 4,800 (41.29%) were low-frequency codes, representing 1,263 unique low-frequency codes. This significant proportion of low-frequency codes, especially in prioritized positions, demonstrates the realistic clinical coding challenges our model was evaluated against.
